# Optimization of UV-Curable Polyurethane Acrylate Coatings with Hexagonal Boron Nitride (hBN) for Improved Mechanical and Adhesive Properties

**DOI:** 10.3390/polym16172544

**Published:** 2024-09-09

**Authors:** Vishal Gavande, Shanmugam Mahalingam, Junghwan Kim, Won-Ki Lee

**Affiliations:** 1 BB 21plus Team, Department of Polymer of Polymer Engineering, Pukyong National University, Busan 48513, Republic of Korea; vgawande77@gmail.com; 2 Department of Materials System Engineering, Pukyong National University, Busan 48513, Republic of Korea; shanmugam82@pknu.ac.kr (S.M.); junghwan.kim@pknu.ac.kr (J.K.)

**Keywords:** hexagonal boron nitride (hBN), coatings, nanocomposites, UV-cured coatings, thermal stability

## Abstract

Polymer coatings are widely used in industries for protection, decoration, and specific applications, typically including volatile organic compounds (VOCs) to achieve low viscosity. The growing environmental concerns and the anticipated limits on fossil feedstock have driven the coating industry towards eco-friendly alternatives, with UV-curing technology emerging as a promising solution due to its energy efficiency, low-temperature operation, reduced VOC emissions, and high curing speed. Polyurethane acrylates (PUAs) are critical in UV-curable formulations, offering excellent flexibility, impact strength, optical, and adhesion properties. However, UV-cured PUA coatings face limitations in thermal stability and tensile strength, which can be addressed by incorporating fillers. This study investigates the effects of multi-functionalized hexagonal boron nitride (hBN) nanoparticles on the mechanical, thermal, optical, and adhesion properties of UV-cured PUA films and coatings for pre-coated metals. The results demonstrated that incorporating hBN nanoparticles enhanced the mechanical and thermal properties of the nanocomposite films, with optimal performance observed at 0.5% hBN loading. Despite the improved properties, the FTIR spectra indicated that the low concentration of hBN did not produce significant changes, potentially due to the overshadowing signals from the difunctional polyurethane acrylate.

## 1. Introduction

Polymer coatings are extensively used in industries for protection, decoration, and specialized applications. Typically, these coatings contain volatile organic compounds (VOCs) to maintain a suitable viscosity for application [1,2,3,4]. However, the anticipated depletion of fossil feedstocks and growing environmental concerns have led to increased costs and stricter regulations in the coating industry. Reducing VOC emissions has become crucial in developing eco-friendly coatings. Traditional methods and eco-friendly alternatives offer distinct advantages and limitations in the realm of coating technologies. Traditional coating technologies often rely on solvent-based systems that, while effective in application and durability, pose significant environmental concerns due to the release of volatile organic compounds (VOCs). These VOCs contribute to air pollution and can have adverse health effects, making them less sustainable in the long term. In contrast, eco-friendly coating technologies, such as UV-curing systems, represent a notable shift towards more sustainable practices. UV-curing coatings utilize ultraviolet light to cure and harden the coating instantly, significantly reducing or eliminating the need for solvents. This process not only minimizes the environmental impact by reducing VOC emissions but also enhances efficiency by shortening curing times and improving overall performance. The shift towards UV-curing technologies highlights a growing emphasis on reducing environmental footprints while maintaining high-quality performance. This transition is crucial as industries seek to balance their operational needs with ecological responsibility, making UV-curing an increasingly significant choice in modern coating applications. UV-curing technology has emerged as a leading green technology, offering advantages over thermal curing such as energy efficiency, low-temperature operation, reduced VOC emissions, and high curing speed [2,5,6]. Polyurethane acrylates (PUAs) are a key class of resins in UV-curable formulations, known for their excellent flexibility, impact strength, optical properties, and adhesion [7,8,9,10,11,12,13]. Despite these benefits, UV-cured PUA coatings face limitations in engineering applications due to poor thermal stability and low tensile strength [14,15,16,17]. These drawbacks can be significantly mitigated by incorporating macroscopic and nanoscopic fillers, enhancing both processing and material properties.

The incorporation of stiff anisotropic nanomaterials within a polymeric matrix has steadily attracted interest due to the ability to tailor not only thermo-mechanical properties but also endow the polymer matrix with other additional features that are of massive significance for applications in some emerging areas. Hexagonal boron nitride (hBN) is highly similar to graphite concerning its structure and has high thermal conductivity, extreme electrical insulation, and a low thermal expansion coefficient [18,19,20,21,22,23]. hBN possesses a flat surface with a plate-like shape corresponding to the basal planes of its hexagonal crystal structure. These basal planes are molecularly smooth and lack functional groups available for chemical bonding or other interactions on the surface [24,25,26]. However, homogeneous dispersion and adhesion of hBN in the PUA matrix interface is significant because it has a high surface energy and amphiphobic nature [1]. To eliminate these drawbacks, many surface modification methods have been used, such as functionalization, acid treatment, and plasma treatment [1,27,28,29].

Most studies on boron nitride (BN) and its composites have focused on enhancing mechanical and thermal properties. Similarly, incorporating hexagonal boron nitride (hBN) into the PUA matrix improves several properties. For example, K. Kim et al. reported that BN-filled UV-cured PUA composites significantly improved mechanical properties and long-term stability for underwater sonar encapsulant applications [1]. H. Liu et al. studied the mechanical and anti-corrosion properties of hBN-modified UV-cured waterborne polyurethane dispersions, noting a twofold increase in stress and a fivefold increase in Young’s modulus compared to unmodified dispersions [30]. In our study, we investigated the effects of modified hBN nanoparticles on UV-cured PUA films and coatings, demonstrating a potentially effective matrix system for pre-coated metals. We examined the mechanical, thermal, optical, and adhesion properties of hBN/PUA nanocomposites in detail, including the adhesion performance of hBN/PUA coatings on pre-coated metal sheets.

## 2. Experimental

### 2.1. Materials

Difunctional polyurethane acrylate oligomer (Miramer PU2100, viscosity 7000 cPs at room temperature, average molecular weight 2100) and isobornyl acrylate monomer (IBOA, Miramer M1140) were supplied by Miwon Specialty Chemicals, Anyang, South Korea. The photocatalyst 1-Hydroxy-cyclohexyl-phenyl-ketone (Irgacure 184D) was obtained from Ciba Specialty Chemicals, Basel, Switzerland. Boric acid and urea were sourced from Sigma Aldrich, St. Louis, MA, USA. Dimethylformamide (DMF), hydrogen peroxide (H_2_O_2_), nitric acid, acetic acid, and ethanol were purchased from Samchun, Pyungtaek, South Korea. Polyester-melamine-based pre-coated metal sheets (black and gray) were provided by Dongkuk Steel Mill, Seoul, South Korea. All chemicals were used without further purification.

### 2.2. Synthesis of hBN Nanosheets

Boric acid and urea were mixed in a 1:24 molar ratio in 50 mL of deionized water under stirring conditions and heated at 70 °C until complete evaporation of the water occurred. The resulting dried white solid mixture was transferred to a ceramic boat and heated at 900 °C for 5 h under a nitrogen atmosphere in a tubular furnace. The sample was then allowed to cool naturally to room temperature, yielding a white, foam-like, highly porous boron nitride. The obtained hBN powder was mixed in dimethylformamide (DMF) and subjected to ultrasonication for 4 h. Following sonication, the hBN suspension was transferred for centrifugation. After centrifugation, the hBN was washed with deionized water to remove any residual solvent and dried at 60 °C for 24 h.

### 2.3. Functionalization of hBN Nanosheets

To functionalize hexagonal boron nitride (hBN) with organic groups, 1 g of hBN powder was dispersed in an oxidizing solution, such as 3% hydrogen peroxide (H_2_O_2_) or 0.1 M nitric acid (HNO_3_). The mixture was ultrasonicated for 1 h and heated at 60 °C for 2 h to introduce oxygen-containing groups. After oxidation, the oxidized hBN was separated by centrifugation, washed with deionized water, and dried at 80 °C overnight. The dried hBN was then dispersed in ethanol, and an organic functionalizing agent like acetic acid in excess was added. This mixture was stirred at room temperature for 24 h. The functionalized modified hexagonal boron nitride (m-hBN) was collected by centrifugation, washed with ethanol and water to remove unreacted agents, and dried again at 80 °C overnight.

### 2.4. Fabrication of m-hBN/PUA Nanocomposites

The UV-curable PUA resin was formulated by combining 50% difunctional polyurethane acrylate, 45% isobornyl acrylate (IBOA), and 5% photocatalyst. IBOA served as a reactive diluent, while Irgacure 184D was used as a photocatalyst to absorb UV radiation and generate reactive species that initiate UV-curing polymerization. Achieving a homogeneous dispersion of the nanoplatelets in the PUA matrix was crucial for enhancing several properties. To prepare the multifunctional hBN nanoplatelets, they were first evacuated at 50 °C in a vacuum oven for 2 h before processing. The nanoplatelets were then incorporated into the PUA at varying proportions (0.1, 0.2, 0.5, 1, 2, and 5 wt %) through the following steps:

1. The m-hBN nanoplatelets were added to IBOA and blended using a vortex mixer.
2. The appropriate amount of PUA and photocatalyst were then added to the mixture, which was blended for 1 h.
3. After mixing, the blend was subjected to ultrasonication for 2 h and subsequently mixed again for 1 h using a vortex mixer.

**Figure 1:**
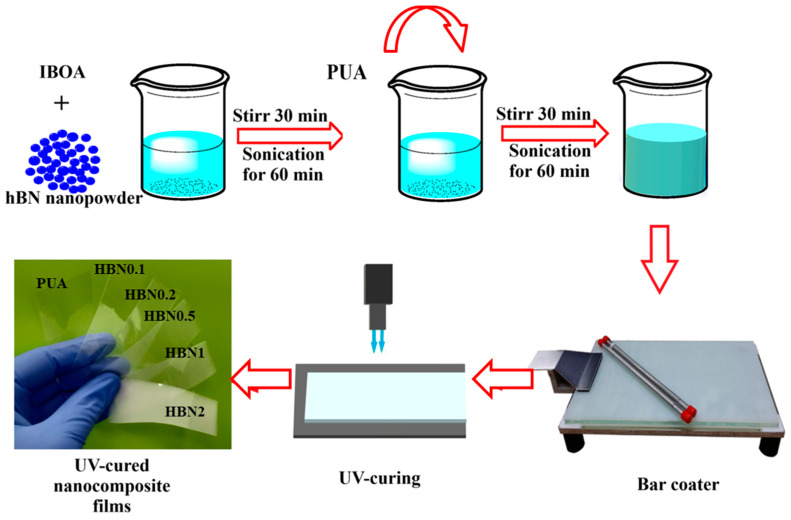
Schematic presentation of UV-curable modified hBN/PUA nanocomposite film fabrication method.

**Figure 2:**
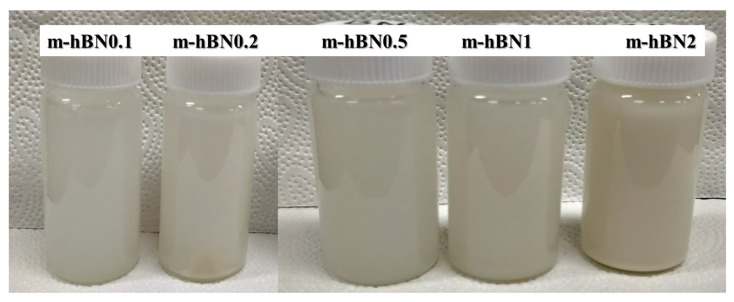
Dispersion of modified m-hBN in PUA formulation after 48 h.

The detailed fabrication method of m-hBN/PUA nanocomposite films is depicted in Figure 1. For the fabrication of neat samples, the designed UV-curable polymer formulation was mixed vigorously using a vortex mixer. All samples were stored in a dark room for 24 h before experimentation to ensure proper dispersion and reaction. The dispersion was homogeneous even after the 48 h of mixing m-hBN in PUA formulation (Figure 2).

Firstly, after degassing, the formulated hBN/PUA mixture was cast on the glass plate using a 200 µm bar coater for films and a 50 µm bar coater for coating on the pre-coated metals and kept in the air-tight metallic mold, which has a quartz plate on the top surface. Afterward, the mold was purged with nitrogen to avoid oxidation during the UV-curing polymerization and transferred to the UV-curing unit. The wet nanocomposite was cured using a conveyor belt-type UV-curing machine (LZ-U101, Lichtzen Co. Ltd., Gunpo, Republic of Korea) equipped with a gallium lamp (160 W/cm, main wavelength: 365 nm, UV-A 1100 mJ/cm^2^, Arc system). The thickness of the hBN/PUA nanocomposite film was between 100 and 120 µm, while the hBN/PUA nanocomposite coating was 30 µm thick.

### 2.5. Characterizations

Fourier transform infrared (FTIR) spectra were recorded using a Thermo Scientific ATR unit (Thermo Scientific Nicolet Is10, Waltham, MA, USA) with a spanning wavenumber ranging from 4000 to 400 cm^−1^ with a resolution of 4 cm^−1^ and 64 scans. Tensile tests (ASTM D638) were conducted on a universal testing machine (Tinius Olsen, H1KT machine, Horsham, PA, USA, equipped with a 100 kgf load sensor) at a crosshead speed of 5 mm/min. Six replicas of each type of nanocomposite film sample were prepared using a dog-bone-shaped mold press with a gauge length of 10 mm and a central width of 2 mm. UV-visible transmission spectra were recorded over a wavelength range of 200 to 800 nm at a 1 nm scan rate using a Hitachi (U-2010) spectrophotometer, Tokyo, Japan. The crystal structure of synthesized hBN and m-hBN was characterized via powder X-ray diffractometry (PXRD) using Cu-Kα radiation (30 kV PANalytical/X’Pert3-Powder), in a 2θ range of 10° to 80°. The thermal characteristics of the PUA films and N6/PUA nanocomposite films were measured in a nitrogen atmosphere at a heating rate of 10 ℃/min by thermogravimetric analysis (TGA 7, Perkin-Elmer, Waltham, MA, USA). The static contact angle of the nylon 6 nanofibrous films was determined using 500 μL syringes with a 0.5 mm needle diameter and 38 mm length at 22 ± 2 °C. For drop shape analysis (KRUSS DSA100, Berlin, Germany), 3 μL volume of epoxy resin was utilized at a rate of 600 μL min^−1^. To capture sharp needle images, a charge-coupled device camera was attached for an ideal image adjustment. In order to achieve an accurate value, five drop shape readings were captured, and the average was determined.

The adhesion properties of hBN/PUA nanocomposite coatings were evaluated by applying them to pre-coated metal sheets. The cross-cut tape test was performed using an adhesion tester (BGD 503 Cross-Cutting Rule, Guangdong, China), creating 100 cross-cut cells spaced 1 mm apart, followed by applying strong adhesive tape four times in each direction. To assess the adhesion properties after deformation, the Erichsen test was conducted using a Cross-cut Erichsen Cupping Tester (BGD 309 Cupping Tester, Guangdong, China). The 100 cells were cross-cut with a 1 mm gap, deformed to 7 mm, and the cross-cut tape test was repeated with strong adhesive tape applied four times in each direction.

The gel content of the cured films was determined by measuring the weight loss after a 48 h immersion in acetone [31]. The specimens were then dried in a vacuum oven at 60 °C and weighed again. Gel content values were calculated from the weight ratio of the specimens before and after immersion.

## 3. Results and Discussions

**Figure 3:**
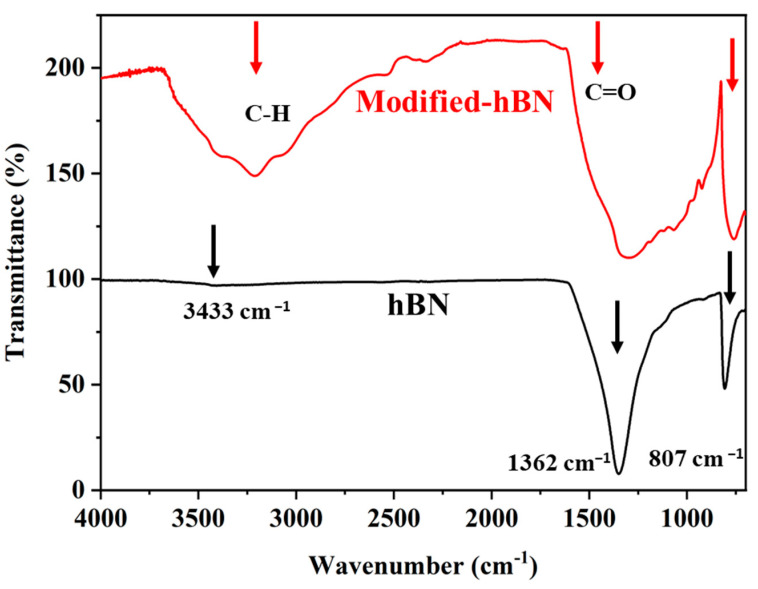
FT-IR spectra of synthesized unmodified and modified hBN.

As shown in Figure 3, the FTIR spectrum of hBN typically shows a strong and broad absorption band around 1380 cm^−1^ and 807 cm^−1^ (displayed with black arrows), which is attributed to the B-N stretching vibrations in the hexagonal lattice structure [32,33,34]. This peak is characteristic of hBN and confirms the presence of the B-N bonds. The FTIR spectrum of m-hBN shows the same characteristic B-N stretching band around 1380 cm^−1^ as hBN, indicating the presence of the hexagonal boron nitride structure. However, the m-hBN spectrum also exhibits additional carbonyl peaks (C=O) in the region between 1500–1700 cm^−1^ and broad peak around 3000 cm^−1^ indicated C-H stretching vibrations confirming the attachments of organic groups (displayed with red arrows). These additional peaks suggest the presence of functional groups or modifications introduced during the modification process of hBN. 

**Figure 4:**
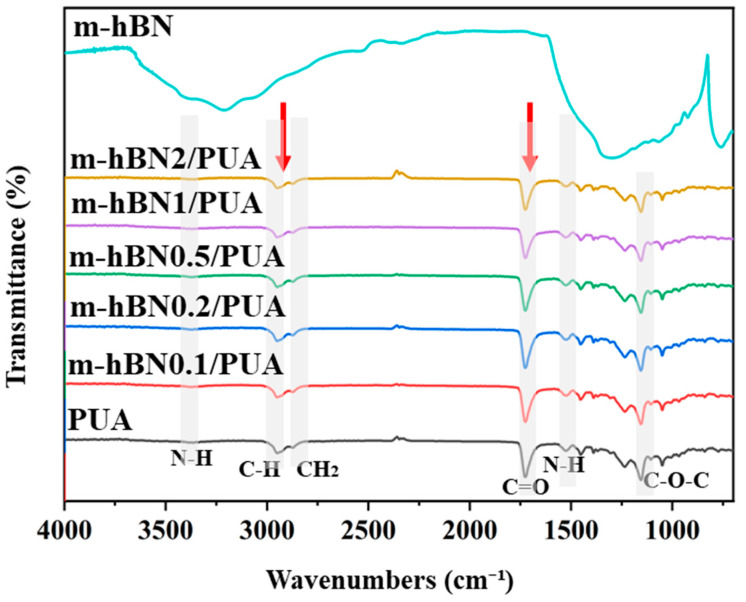
FT-IR spectrums of PUA, nanocomposite films, and m-hBN.

Figure 4 illustrates the FTIR spectrum of m-hBN/PUA nanocomposite films measured in ATR mode to confirm photopolymerization. In the cured PUA film spectrum, the acrylate group (C=C) peaks at 1635 and 810 cm^−1^ disappeared due to the crosslinking reaction from photopolymerization (Figure 3) [2]. The FTIR spectrum of pure PUA exhibited typical bands corresponding to the hard segments: N-H (1528, 3334 cm^−1^), C=O (1727 cm^−1^), and C-O-C (1242 cm^−1^) groups, as well as soft segments C-H and CH_2_ (2870 and 2950 cm^−1^) groups (grey highlighted columns in Figure 4). However, the spectrum of hBN/PUA nanocomposite films showed no obvious peaks of m-hBN. This suggests that the amount of modified hBN (2%) might be too low to produce significant changes in the FTIR spectra of the nanocomposite [35,36]. The dominant signals from the difunctional polyurethane acrylate could overshadow those of the modified hBN, especially if the modifications to the hBN are subtle or the functional groups are present in low concentrations. Additionally, the characteristic peaks of the modified hBN might overlap with those of the difunctional polyurethane acrylate, making their signals indistinguishable if the functional groups on the hBN are similar to those in the polymer matrix.

**Figure 5:**
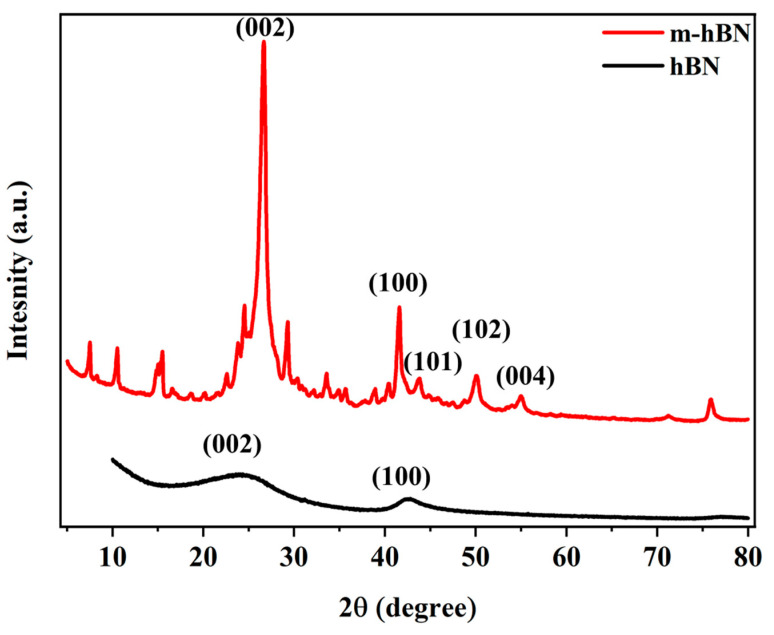
XRD spectra of hBN and m-hBN powder samples.

The XRD pattern of hBN confirms the presence of a well-defined hexagonal structure, as expected from the FTIR analysis. As shown in Figure 5, the XRD patterns for both hBN and m-hBN show prominent peaks at specific 2θ angles, indicating the presence of crystalline structures. The major peaks for hBN appear at approximately 26° and 43°, corresponding to the (002) and (100) planes, respectively [36,37]. These positions are typical for hexagonal boron nitride (hBN). The m-hBN sample shows additional peaks compared to hBN, suggesting structural modifications. These new peaks are observed at around 34°, 41°, and 50°, corresponding to the (101), (102), and (004) planes, respectively [38]. The introduction of functional groups (carbonyl and C-H) through organic modification is likely to disrupt the regular arrangement of atoms in the hBN lattice. The peak intensities of m-hBN differ from those of hBN, indicating changes in the crystallographic orientation or preferred orientation of the modified material [39,40].

**Figure 6:**
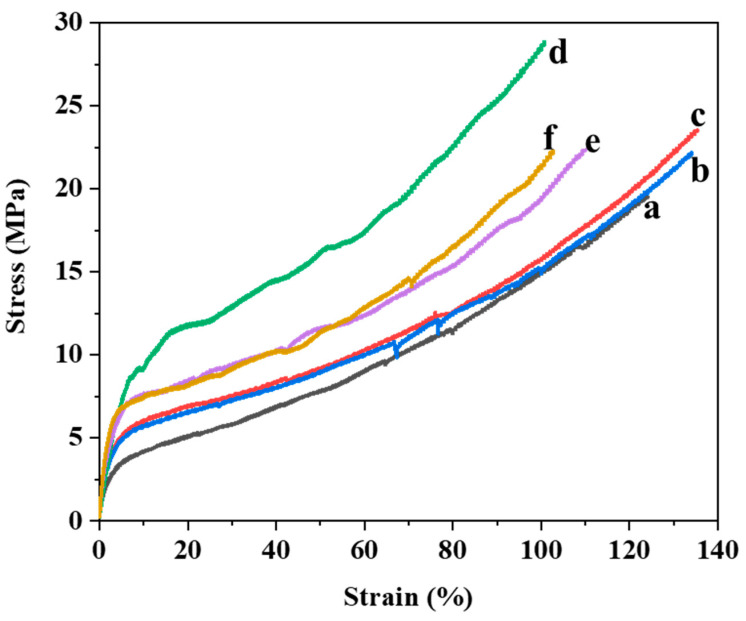
Examples of stress-strain curves of (a) PUA, (b) m-hBN0.1PUA, (c) m-hBN0.2PUA, (d) m-hBN0.5PUA, (e) m-hBN1PUA, and (f) m-hBN2PUA nanocomposite films.

Figure 6 shows the tensile behavior of the PUA films and hBN/PUA nanocomposite films. The PUA films exhibited an average tensile strength at break of 17.50 ± 1.23 MPa and an elongation of 94.85 ± 8.38%. As listed in Table 1, the tensile strength and elongation of the hBN/PUA nanocomposite films (0.1%, 0.2%, and 0.5%) increased with the addition of hBN but later decreased as the hBN content reached 1% and 2%. Specifically, the tensile strength at break for the hBN nanocomposite films improved incrementally to 20.93 ± 0.92 MPa, 23.07 ± 1.35 MPa, and 26.37 ± 2.12 MPa as the hBN content increased to 0.1%, 0.2%, and 0.5%, respectively. However, it decreased to 22.64 ± 1.07 MPa and 21.67 ± 0.76 MPa as the hBN content increased to 1% and 2%, respectively (Table 1).

The addition of modified hBN nanoplatelets to the PUA matrix significantly enhanced the tensile strength due to the higher rigidity of the inorganic fillers compared to the reference PUA. The tensile strength initially increased including Young’s modulus with the addition of hBN nanoparticles but showed a downward trend beyond 0.5% hBN content. Young’s modulus of the m-hBN/PUA nanocomposite generally increases as the m-hBN content increases. This trend supports the idea that m-hBN effectively reinforces the PUA matrix. This means the addition of m-hBN to PUA significantly enhances its Young’s modulus, making it a stiffer material. This improvement is primarily attributed to the reinforcement effect of m-hBN and its effective dispersion within the PUA matrix. The robust adhesion of the nanoparticles with the matrix material was crucial for the tensile strength of the nanocomposites. Better interfacial adhesion and homogeneous dispersion between the multifunctional hBN nanoplatelets and the PUA matrix reduced stress concentration areas in the nanocomposite films. The optimal mechanical properties were observed at the 0.5% m-hBN/PUA nanocomposite film. The reinforcement provided by modified hBN nanoplatelets allowed for the absorption of fracture energy and retardation of crack propagation [6]. The decline in mechanical properties with higher hBN content (1% and 2%) can be attributed to the excessive loading of modified hBN, which leads to partial aggregation. This aggregation results in insufficient interaction with the PUA matrix, creating stress concentration areas and consequently reducing mechanical properties [41,42,43,44].

**Figure 7:**
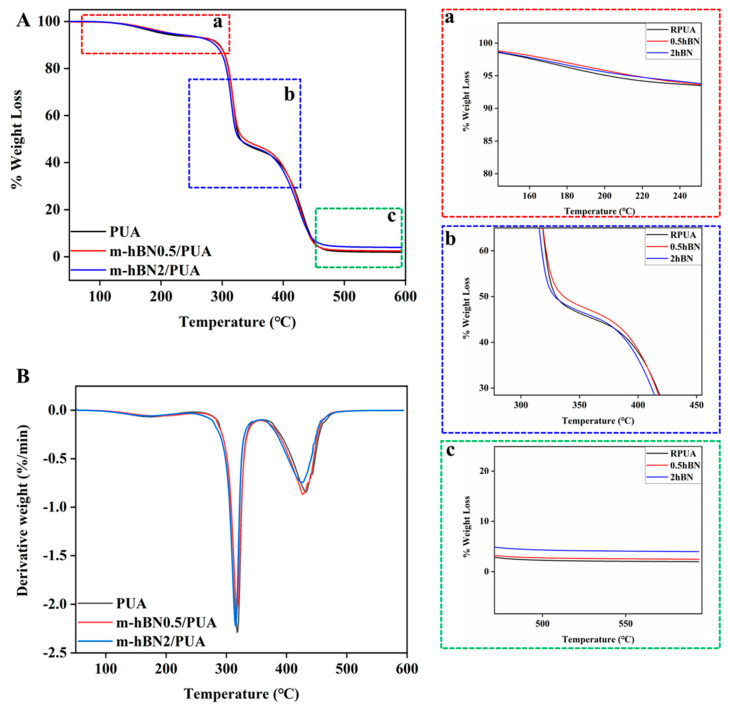
TGA (**A**) and DTG (**B**) thermograms of the UV-cured films: (a), (b), and (c) are the zoomed images of respective sections.

The thermogravimetric analysis test was performed, and the thermal properties of PUA, 0.5%, and 2% hBN/PUA nanocomposite films were investigated. The experiments were conducted up to 600 °C under a nitrogen atmosphere at a heating rate of 10 °C min^−1^. Figure 7 depicts that decomposition was observed in mainly three steps, and weight loss was observed of about 5%, 10%, and 50% for PUA, 0.5%, and 2% hBN/PUA nanocomposite films. All of the films show three shoulder peaks, and the maximum decomposition rate occurs at 160 °C in the first stage due to the decomposition of urethane bonds in hard segments. As shown in Figure 7A-a, the decomposition temperature of the 0.5% and 2% hBN/PUA nanocomposite films were found to increase due to the addition of hBN nanoplatelets, which shows higher decomposition temperatures of 215 and 214 °C, respectively, for 5% weight loss (Table 2). The second stage organizes from 290 to 325 °C, and the maximum decomposition rate was at 310 °C, which results from the decomposition of the soft urethane segments (Figure 7A-b). The decomposition temperature at 10% weight loss of the 0.5% and 2% hBN/PUA nanocomposite films were 291 and 288 °C, respectively, compared to reference PUA film (290 °C), and suggested that the film exhibits similar thermal behavior. However, the corresponding temperature at 50% weight loss for the reference PUA film was 330 °C, whereas for both 0.5% and 2% hBN/PUA nanocomposite films were 336 and 328 °C, respectively. In the last stage, the maximum degradation rate was at 425 °C, and substantially the matrix started to decompose, which resulted in a weight loss of 90% (Figure 7A-c). Reinforcement of hBN nanoplatelets does not emerge to have any adverse impact on the decomposition rate. These results indicate that the hBN/PUA nanocomposite films had better thermal stability at the initial stage of decomposition than the PUA films.

The DTG peaks (Figure 7B) represent the decomposition stages of the hard and soft segments in the PUA and hBN/PUA nanocomposite films. The first peak (~160 °C) corresponds to the decomposition of the hard segments (urethane bonds), while the second peak (~310 °C) is associated with the decomposition of the soft segments (polyol chains). The third peak (~425 °C) represents the final breakdown of the polymer matrix [45,46]. The addition of hBN nanoplatelets increases the decomposition temperature of the hard segments, indicating enhanced thermal stability in the nanocomposite films.

As the amount of modified hBN in the PUA matrix increases, the water contact angle generally increases (Figure S1). This suggests that the addition of hBN makes the surface less hydrophilic (less water-wetting). Modified hBN is known to be a hydrophobic material. Its incorporation into the PUA matrix likely imparts its hydrophobic properties to the surface, leading to the observed increase in contact angle. The modification of hBN might introduce functional groups onto its surface, which could repel water molecules. Also, the addition of hBN particles might introduce surface roughness, which can also contribute to increased contact angle due to the trapping of air pockets. The interaction between hBN particles and the PUA matrix might influence the surface energy and hydrophilicity of the nanocomposite film.

**Figure 8:**
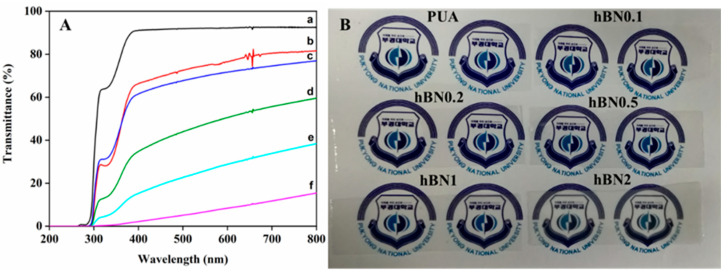
(**A**) Optical light transmittance of (a) PUA, (b) m-hBN0.1PUA, (c) m-hBN0.2PUA, (d) m-hBN0.5PUA, (e) m-hBN1PUA, and (f) m-hBN2PUA nanocomposite films. (**B**) Optical image of the of PUA and nanocomposite films.

The UV-vis spectra of the UV-cured hBN/PUA nanocomposite films with varying amounts of hBN are presented in Figure 8A. The PUA film demonstrated good optical transmittance in the visible range, approximately 91% at 400–500 nm. However, it was observed that increasing the amount of hBN adversely affected the optical transmittance of the PUA films, which is consistent with the addition of nano-sized inorganic fillers [5,8]. In the visible region, the transmittance values decreased after introducing the hBN nanoplatelets in the PUA films. The hBN/PUA nanocomposite films with 0.1%, 0.2%, 0.5%, 1%, and 2% have a transmittance of 70%, 67%, 41%, 20%, and 5%, respectively. Additionally, as shown in Figure 8B, we also ensured the transparency of the nanocomposite films, which represent and are evidence of the significant character of the nanocomposite films.

The gel content of the cured films was examined to evaluate the amount of insoluble parts in the cured coatings. As seen in Table 1, the incorporation of hBN nanoplatelets into the PUA did not affect the curing kinetics, with gel content values ranging from 97–98%, suggesting that the hBN/PUA films were well crosslinked.

**Figure 9:**
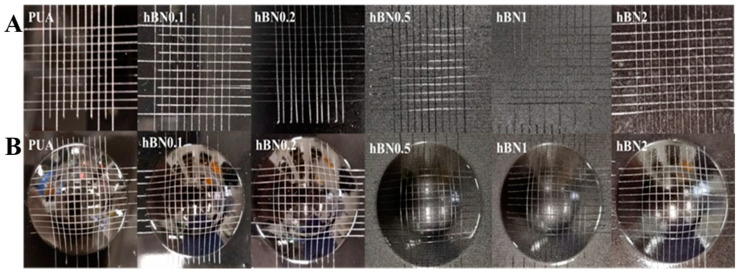
(**A**). Pictorial presentation of cross-cut tape test (before deformation) of the cured coatings. (**B**). Cross-cut Erichsen cupping test (after deformation) of the cured coatings.

To evaluate the adhesion of the coatings, an adhesion test was commonly applied using a tool to cut a right-angle lattice pattern onto the coated substrate [47,48]. PUA and hBN/PUA nanocomposite formulations were cast on polyester-melamine-based colored metal sheets using a bar coater. After UV curing, the coatings were cross-cut into 100 cells with a 1 mm gap, as shown in Figure 9A, and tested with 3M PET tape applied four times in each direction. All cut cells of the coatings in each sample maintained their original status after the tape test. Similarly, to examine the adhesion of the coatings after deformation, the Erichsen test was conducted using an Erichsen cupping tester. After cross-cutting the coatings into 100 cells with a 1 mm gap, they were deformed to 7 mm. Subsequently, the cross-cut test was applied using 3M PET tape (St. Paul, MN, USA) four times in each direction. The standard deformation index in pre-coated metal coating industries is 6 mm, where no cells should peel off after 6 mm of deformation and cross-cut testing. As shown in Figure 9B, PUA and hBN nanocomposite coating samples demonstrated deformation up to 7 mm without any trace of cracks. Additionally, there was no impact on any of the coatings after 7 mm of deformation, and they remained unchanged.

The incorporation of inorganic nanoparticles into the matrix can reduce impact properties. However, by adding hBN nanoplatelets to the coatings, we aimed to avoid lowering toughness while enhancing the mechanical and thermal properties.

## 4. Conclusions

In this study, modified hexagonal boron nitride (hBN) nanoparticles were successfully incorporated into UV-curable polyurethane acrylate (PUA) to enhance the properties of the resulting nanocomposite films and coatings. The addition of hBN significantly improved the tensile strength and thermal stability of the nanocomposite films, with the optimal mechanical properties observed at a 0.5% hBN loading. The FTIR analysis confirmed the photopolymerization process but did not show significant peaks for hBN, suggesting that the low concentration of hBN was overshadowed by the dominant signals of PUA or overlapped with them. The thermal analysis revealed improved thermal stability in the initial decomposition stages for the hBN/PUA nanocomposites. Furthermore, the optical transmittance decreased with increasing hBN content, indicating the impact of inorganic fillers on the transparency of the films. Adhesion tests demonstrated excellent performance, with no peel-out observed even after deformation, highlighting the potential of hBN/PUA nanocomposite coatings for pre-coated metal applications. Overall, the incorporation of hBN nanoparticles into PUA matrices presents a promising approach to enhance the properties of UV-curable coatings, making them suitable for various industrial applications.

## Figures and Tables

**Table 1:** Mechanical properties and % gel content of the cured m-hBN/PUA nanocomposite films.

Sample	Ultimate Tensile Strength (MPa)	Elongation @ Break	Young’s Modulus (MPa)	Gel Content (%)
PUA films	17.50 ± 1.2	94.85 ± 8.3	121.38 ± 9.8	98.1
m-hBN0.1/PUA	20.93 ± 0.9	119.33 ± 8.1	168.76 ± 12.3	97.9
m-hBN0.2/PUA	23.07 ± 1.3	130.89 ± 8.1	166.70 ± 6.3	98.5
m-hBN0.5/PUA	26.37 ± 2.1	113.16 ± 5.9	178.72 ± 15.8	97.3
m-hBN1/PUA	22.64 ± 1.0	110.16 ± 3.6	213.93 ± 10.0	98.0
m-hBN2/PUA	21.67 ± 1.7	116.95 ± 8.5	240.13 ± 22.1	97.5

**Table 2:** TGA results for the cured films with different loading of hBN nanoplatelets.

Sample	T_5%_ Weight Loss (°C)	T_10%_ Weight Loss (°C)	T_50%_ Weight Loss (°C)	Char Residual at 600 °C (Weight %)
PUA film	201	290	330	1.96
m-hBN0.5/PUA	215	291	337	2.45
m-hBN2/PUA	214	288	329	3.96

## Data Availability

The data presented in this study are available on request from the corresponding.

## Supplementary Materials

The following supporting information can be downloaded at: https://www.mdpi.com/article/10.3390/polym16172544/s1, Figure S1: Examples of Water Contact angles of (a) PUA, (b) m-hBN0.1PUA, (c) m-hBN0.2PUA, (d) m-hBN0.5PUA, (e) m-hBN1PUA, and (f) m-hBN2PUA nanocomposite films.
