# Supplementary material for: Optimization of UV-Curable Polyurethane Acrylate Coatings with Hexagonal Boron Nitride (hBN) for Improved Mechanical and Adhesive Properties
Source: Polymers (Basel). 2024 Sep 9;16(17):2544. doi: 10.3390/polym16172544 (PMC11398060; doi:10.3390/polym16172544)
Supplement: Supplementary file 1 [file polymers-16-02544-s001.zip › polymers-3160166-supplementary.pdf]

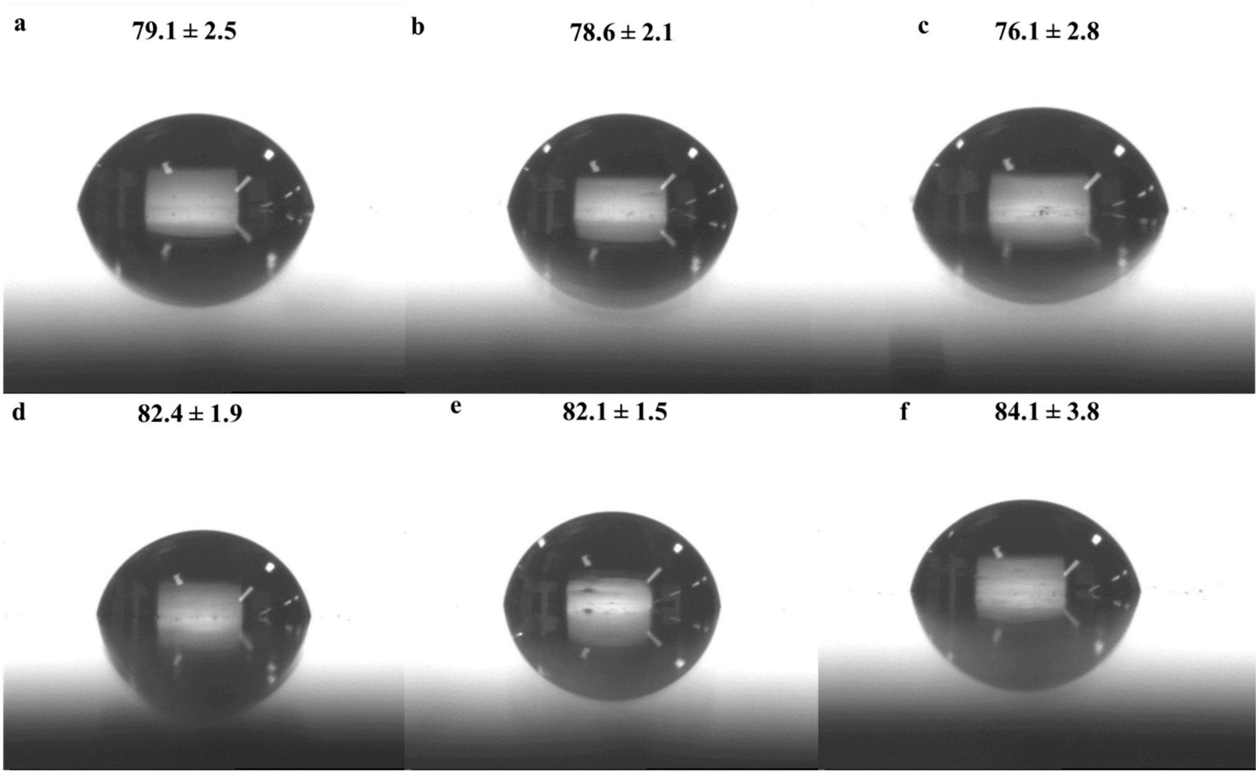

Figure S1. Examples of Water Contact angles of a) PUA, b) m-hBN0.1PUA, c) m-hBN0.2PUA, d) m-hBN0.5PUA, e) m-hBN1PUA, and f) m-hBN2PUA nanocomposite films.
